# Identifying the Symptom Severity in Obsessive-Compulsive Disorder for Classification and Prediction: An Artificial Neural Network Approach

**DOI:** 10.1155/2020/2678718

**Published:** 2020-06-22

**Authors:** Mirza Naveed Shahzad, Muhammad Suleman, Mirza Ashfaq Ahmed, Amna Riaz, Khadija Fatima

**Affiliations:** ^1^Department of Statistics, University of Gujrat, Pakistan; ^2^Department of Management Sciences, University of Gujrat, Pakistan

## Abstract

The present study is aimed at identifying the most prominent determinants of OCD along with their strength to classify the OCD patients from healthy controls. The data for this cross-sectional study were collected from 200 diagnosed OCD patients and 400 healthy controls. The respondents were selected through purposive sampling and interviewed by using the Y-BOCS scale with the addition of a factor, worth of an individual in his family. The validity and reliability of data were assessed through Cronbach's alpha and confirmatory factor analysis. Artificial Neural Network (ANN) modeling was adopted to determine threatening determinants along with their strength to predict OCD in an individual. The results of ANN modeling depicted 98% accurate classification of OCD patients from healthy controls. The most contributing factors in determining the OCD patients according to normalized importance were the contamination and cleaning (100%); symmetric and perfection (72.5%); worth of an individual in the family (71.1%); aggressive, religious, and sexual obsession (50.5%); high-risk assessment (46.0%); and somatic obsessions and checking (24.0%).

## 1. Introduction

Obsessive-compulsive disorder (OCD) is a common mental and behavioral disorder recognized by the presence of obsessions (frequent unwanted sensations) and compulsions (recurring behaviors considered compulsive to perform) in the patients [1]. The most common obsessions are thoughts of religious obsessions, dirt and contamination, sexual thoughts, perfectionism, fear of insecurity, particular ordering of things, and such similar obsessions. The common known compulsions are the excessive checking, cleaning, washing, arrangement, and order and other related activities patients feel the urge to perform to avoid obsessions. Generally, people suffering from OCD spend much time performing undesired and uncontrolled behaviors to deal with their obsessions. These repeated behaviors affect the daily routines by consuming a considerable amount of patient's time. Furthermore, the patients remain mentally engaged by such unwelcoming and irritating thoughts that cause problems not only for patients but also for their families too. Epidemiological studies have reported the worldwide prevalence of OCD between 1 and 3% across all genders and age groups [2, 3]. The World Health Organization (WHO) has marked such mental diseases among the top ten leading causes of death worldwide [4]. It is also known as a serious cause of increasing suicidal attempt rate [5] and disability of work. The national comorbidity survey replication has also revealed OCD as a leading cause of mental anxiety disorder that has serious implications [3]. Recently, the emerging behaviors related to excessive use of social media, online video games, mobile usage, and selfies phobia are becoming the reasons for changing brain and behaviors in the youth [6]. Similarly, the feeling of insignificance or negligence in social networks is regarded as an important cause of OCD in many cultures. Therefore, patients suffering from OCD seriously need awareness, diagnostic, and timely treatment before leading to a chronic disability.

Evidence about the prevalence of OCD can be traced from ancient times. However, people of that time were not aware of its actual causes and believe that people suffer from OCD due to religious or spiritual causes [7]. It was firmly believed, even in the 6^th^ century, that persons with OCD have blasphemous beliefs about religion and fears of perdition by lord [8]. The modern terminologies of this psychiatric disorder were introduced in medieval times [9, 10], and most of the present terms are originated from Latin. Later, OCD was considered as a mental disorder that strongly affects the emotional states of a person. Varieties of OCD's symptoms are pointed out since 1830 including compulsions and fear of contamination as the prominent among them. Pierre Janet and Sigmund Freud were the first to describe OCD as something separate from neural disorder. Freud argued that such kinds of disorders can be the result of the imbalance between unconscious sexual impulses, conscience, and reality [11]. He further noticed these obsessions and compulsions as a defense mechanism against overwhelming and emotionally charged material. Despite these OCD-related developments, the physical, psychological, or even pharmacy-related treatment options have remained insufficient for offering a proper treatment of obsessive and compulsive disorders [12].

Many scales have been developed to measure the presence and severity of OCD. One of the most popular and comprehensive scales for OCD measures is the Y-BOCS with excellent validity and reliability [13]. It was developed in 1986 to assess the symptom severity and outcome in OCD treatment. Still, Y-BOCS is the most common measure of OCD severity and it is considered as the gold standard and progressively used in clinical trials [14]. It is used to determine the presence of different types of obsessions and compulsions. In this scale, the first section deals with detailed demographic information that was significantly related to OCD patients. The second section consists of six factors; the first one is a self-created factor that is added according to the situation, namely, “worth of an individual in a family (worth in family).” Waters and Barrett [15] determined that the family factors associated with OCD, and Wilcox et al. [16] accumulated many evidences that parenting and family environment contribute to the development of OCD. It is also explored in many other studies that the family factors are the possible risk factors in the development of OCD, including parental modeling, parental blame, family conflict, family accommodations, and parenting styles [15, 17, 18]. And cultures having a joint family system play a vital role in the development of OCD [19]. Therefore, these and many other studies became the main motivation to consider the “worth in family” factor in this study, as it needs to evaluate in our culture. The factor “worth in family” included variables related to the following: warm and intimate times together with family, feel easy going and relaxed with elders in the family, family conflicts, quarreling and antagonism family environment, get sympathy when upset or frustrated, allows give input into family rules, can freely express myself with parents/partner and siblings, get good respect on my opinions, facing authoritarian elders' style, living in the restrictive family environment, and presence of psychological disorder in any family member. The remaining five factors of Y-BOCS were factorized by Denys in 2004 [20], named as contamination and cleaning; aggressive, sexual, and religious obsession; somatic obsessions and checking; symmetric and perfection; and high-risk assessment which is the sixth factor. All the variables in each factor were measured on a five-point Likert scale.

Nonclinical studies are good enough in understanding, maintaining, and dealing with OCD [21], though international researchers have defined, analyzed, and described multiple modified and nonmodified determinants of OCD. However, in the literature, very few researches are found about the classification of the case-control data on the base of prominent determinants to determine the presence or absence of OCD. The aim of this study was, therefore, to diagnose the potential determinants of OCD with their strength of importance by employing Artificial Neural Network (ANN) in case-control study samples to distinguish between OCD patients and healthy controls. Consequently, the identification of OCD patients is the main focus of interest and substantial investigation. We believe, by using this strategy of analysis, we can produce easier, authentic, and parallel to the clinically relevant results. In Pakistan, there is a need for developing more insight into the existence of this disorder as it is mostly underrecognized and undertreated [22]. Identifying the determinants of OCD with their magnitude will be helpful for both psychiatrists and family practitioners.

## 2. Subjects and Methods

It was a cross-sectional study conducted in two cites (Gujrat and Rawalpindi) of Punjab, Pakistan. In the present study, a purposive sampling technique was used to interview OCD patients and healthy controls. The rationale of choice of this sampling technique was based on the consideration that it is a psychiatric disease and patients are only available in the psychiatric clinic and hospitals. The sample size was calculated based on Cochran provided formula [23] (*n* = *Z*^2^*pq*/*e*^2^) that allows calculating an ideal minimum sample size from the unknown population with a desired level of precision (*e*), confidence level (*Z*), and the estimated proportion ($\hat{p}$) of the attribute present in the population. In this study, according to the experts' opinion, 5% OCD cases proportion in a population were considered, with *e* = 3% and *Z* = 95%. Using these values in Cochran's formula, the minimum sample size 202 was obtained. Hence, in this study, 202 OCD cases were interviewed but 2 filled questionnaires were discarded due to incomplete information. Therefore, a double of OCD cases, 400 healthy controls were selected for the interview. The OCD case group participants were recruited through psychiatrist and physician referrals following DSM-IV TR (2000) criteria and were treating as OCD patients.

The data was collected through personally administered questionnaires with the help of two enumerators (one male and one female) who were trained by an experienced psychiatrist. The male and female respondents were approached and assisted by the same gender enumerator. The respondents with medical illness or disability addition to OCD were excluded from the case group. For this purpose, the enumerators visited different hospitals and psychiatry clinics with their consents. The purpose of the research was briefed to the participants or the individuals accompanying them, and they were assured about the confidentiality of the provided information. The unit of analysis was each participant, and responses were recorded individually. The desired task was time taking and difficult to trace out patients of OCD, to approach them, to seek their consent, and finally to get a response from them. The healthy control group participants of this study were selected from a cohort of healthy relatives who had no history of psychiatric or physical disorder and had similar culture and community with the OCD patients. The individual in the control group was excluded if s/he had a psychiatric disorder in his/her lifetime. Both patients and controls' data were collected to ensure transparent evaluation and classification.

The data was analyzed via R-language and SPSS. As gender, age, and community were matched, therefore, these variables were excluded in between-group analyses. The reliability and validity of the scale and factors were tested through Cronbach's alpha and confirmatory factor analysis (CFA). ANN was used to classify the OCD and healthy subject and to obtain the strength of every considered factor in OCD development. ANN is considered as the best tool due to its power, flexibility, the convenience of usage, and cost-effectiveness. It is a statistics handling pattern that is motivated by the method of natural nervous systems. The formation of ANN involves three layers, which are the input layer, hidden layer, and output layer. The number of input and output layers' neurons depends on the study in hand, but the number of neurons in the hidden layer was determined through trial and error; currently, no study exists that suggests the optimal number of hidden layer neurons [24]. The researchers typically divide the dataset into two subsets; first is a training set sample, which helps to establish the network using various weights in the hidden layer, and the second is the testing subset sample, to observe the successfully trained network to classify and to observe the prediction accuracy of new case based on its training. Here, the ANN training set model has been trained by a randomly selected subset of almost 70% of the total sample size (600) while the remaining is used to test the established model. The training model was run until 0.001, or an increasing average square error was found. The performance of the trained model was evaluated on the testing dataset.

## 3. Results

According to the DSM-IV-TR criteria, 200 firmly diagnosed OCD patients and two times more age, gender, and environment almost matched healthy controls were interviewed by Y-BOCS. The demographic information of all the participants is provided in Table 1. In a sample of 600 participants, 42.2% were men, 57.8% were women, and most of them are married (50.5%). The result shows that 58.3% of participants were living in a joint and 41.7% in a nuclear family system. Most of the respondents were living in a middle level of income (43.8%), 36.8% in a low level of income, and few percentages of the respondents were living with a reasonable income level. The age ranged from 12 to 62 of OCD subjects and 16 to 70 of healthy controls with a mean of 31.0 and 32.05 years, respectively. The age of OCD patients, healthy controls, and combined was normal but graphically slightly positive skewed observed. The chi-square test of association was employed to test the association between subjects (categories: OCD and healthy control) and with the categories of gender, family income, marital status, and family structure and found no association between them.

The included factors were highly reliable as Cronbach's alpha values for “worth in family” was 0.74; contamination and cleaning were 0.85; aggressive, sexual, and religious obsession was 0.71; somatic obsession and checking was 0.77; symmetric and perfections were 0.82; and high risk of assessment was 0.86. Moreover, the overall value of Cronbach's alpha of the scale was 0.91. CFA results showed that all factors are significant and followed the recommended goodness of fit indices. This significance was achieved by reducing the number of items; those were insignificant from the relevant factor. Particularly, the discorded items were about sexual behaviors, as the people hesitate in our culture to respond against such items.

To mature the ANN model for the objective of the study, several networks have been evaluated based on an optimized backpropagation type of ANN using logistic activation function (sigmoid), which is most appropriate for binary outcome variable [25]. The final ANN model applied here contained an input, a hidden, and an output layer. The input, hidden, and output layers contained six, four, and one neuron, respectively. The six input neurons (factors) are cleaning and contamination; symmetry and perfection; aggressive, sexual, and religious obsession; high risk of assessment; checking and somatic obsession; and worth in family; those were confirmed by CFA and selected as input variables.

As mentioned earlier, the output variable has two categories; one is the classified OCD subject, and the other is healthy control. The results mentioned in Table 2 show that the overall correctly classified subjects are 98%. The results depict that in the training sample, 95.7% were correctly classified as OCD subjects and 98.3% healthy controls as it was. Only 6 (out of 139) and 5 (out of 290) OCD subjects are declared by the model as healthy control and vice versa, respectively. In the testing part of the sample, there is an overall 98.3% accurate prediction obtained. Therefore, ANN modeling is a valid solution approach in the classification of the disorder.

**Figure 1:**
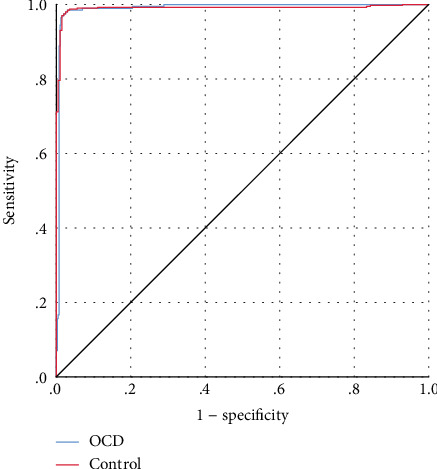
ROC curve for OCD and healthy subjects using ANN; the area under the curve is 0.991.

Additionally, the Receiver Operating Characteristic (ROC) curve shows a visual display of sensitivity and specificity that expresses the classification performance of a model. The area under a ROC curve specifies how well a prediction model differentiates between OCD and healthy subjects. The value of a ROC curve varies between 0.5 and 1.0, which means random guess to perfect accuracy. In Figure 1, the 45-angle line represents the random guessing of the classification. The more the curve moves away from the 45 angle, the more accurate is the classification. Herein, in this study, the ROC curve both lines are in the upper left corner and both wrapping almost all the area. In other sense, the area under the ROC curve is 0.991, which is very close to 1. Consistent with many other studies, the findings of the present study indicated the most crucial threats in the OCD occurrence. Therefore, ANN modeling is a reliable and effective way of predicting the OCD individual.

**Figure 2:**
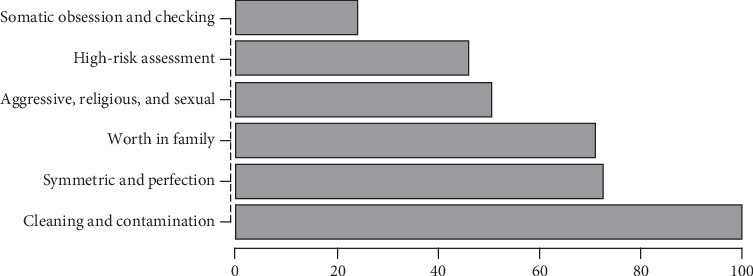
Graphical representation of normalized importance of a predictor.

The severity (importance) of each factor in classification is obtained by the ANN normalized importance graph; the more the strength of a factor means that the factor contributes much to determine the OCD occurrence in an individual. The presentation in Figure 2 shows that the factor cleaning and contamination have the greatest strength in OCD development and to classify an individual as an OCD patient or healthy control. The second highest strength is shown by symmetry and perfection. It is also observed that the “worth in family” has more strength as compared to the aggressive, sexual, and religious obsession; high-risk assessment, and checking and somatic obsession factor. It is evident that an individual comes from the same culture as has the target population with the good worth in family has the least chances of OCD.

## 4. Discussion

This study examined five popular symptom factors of OCD considered in Y-BOCS with the addition of the factor “worth in family.” An ANN approach was used to determine the symptoms that best explained the presence of OCD in patients with their severity using case-control data.

The present research explored that most of the patients of OCD have both obsessions and compulsions and then included only those in the sample data. The findings of the present study reveal that the prevalence of obsessions related to cleaning and compulsion to contamination was the highest, the same as approved on a similar population [26, 27]. The other many related studies also highlighted that the most common symptom is the cleaning or checking compulsions [28]. The results of the present study indicated that the second most common symptom in OCD is symmetric and perfection. The newly added symptom “worth in family” significantly gets the third higher strength in OCD patients. Despite their overt significance, the worth in family is in favor of not happening OCD; therefore, there is an inverse relationship of this factor with OCD. This factor is added with the recommendation and then the consensus of the practitioners of OCD, as they observed and concluded in their years of practice that the patients of OCD get less importance and say in the family in their earlier life. The significant items in the factor “worth in family” were about the worth of your opinion in family matters, freedom to do decision in his/her matters, family members that are supportive and ready to listen to your feelings or problems, the individual being encouraged by his/her positive acts, having other members ready to listen to him/her in a family gathering and can easily spend time with, getting the same importance in the family like siblings, and having the feeling of nervousness in the presence of the elder. The individual items were highly significant and cumulatively the factor that has a substantial adverse effect on the occurrence of OCD. As the case group got less importance in their earlier life than the control group, the more ignorance, disrespect, and badly treated individuals by the family members get more chances of this illness.

In severity, the fourth symptom factor was religious and sexual behaviors. The religious behaviors leave a substantial effect on OCD [29, 30]. As in human life, there is a considerable influence by the religion he/she keeps due to what the religion symbolizes, major faiths, and performing particular acts. Such as numerous studies have been concluded, religiosity beliefs positively correlated with OCD [31]. We could add only one question about sexual behaviors as the people hesitate to answer personal sexual questions. Due to fear of factitious response to the other questions, it is therefore conceivable that these items of the Y-BOCS related to sexual behavior were excluded. The remaining two factors, high-risk assessment and somatic obsession, played a significant but comparatively less role in the classification of two groups (case and control). Notably, nonsignificant gender differences were observed in all symptom factor severity.

The findings of the present study are somewhat similar to the other researches in addition to the “worth in family.” On the base of considered factors, this study truly classified 98% OCD patient and healthy control as reported. Consequently, our findings add a valuable source of information about the dimensional structure of this disorder and provided the comprehensive model for classification of case and control individual.

This study has a few limitations and further research directions. First, some psychiatrists did not allow us to contact their OCD patients that lengthen the process. However, the research can be effectively completed with minimum resources if the government health department owns this study and bound their psychiatrists to cooperate with the researchers. Second, the OCD patients were properly recruited through their diagnostic history, but the healthy controls were the self-reported healthy persons without any known disorder. However, in future studies, healthy persons should be recruited only when they are declared healthy by the psychiatrists after proper diagnostic examinations.

## 5. Conclusions

The findings affirmed that the ANN modeling approach is a good and applicable method to analyze and model the factors affecting the mental health of an individual and predicting the severity of occurrence. All factors were found consistent with previous literature, but their intensity varies. Furthermore, the results indicated that cleaning and contamination factors have the highest strength, and somatic obsession and checking factors have the least strength than other factors. The factor “worth in family” also showed strong significance and strength in OCD. The variation in the strength of the factors of symptoms for OCD than existing studies is due to the deviation in religious, cultural, and social background. Therefore, health decision-makers should plan comprehensive health programs that focus on all of the aforementioned factors in our culture.

## Figures and Tables

**Table 1:** Demographic details of the case (patient), control (healthy), and the total sample.

Variable	Case	Control	Total
Gender	Frequency (%)^∗^	Frequency (%)	Frequency (%)
Male	89 (44.5)	164 (41.0)	253 (42.2)
Female	111 (55.5)	236 (59.0)	347 (57.8)
Family income (in PKR)			
≤20,000	55 (27.5)	166 (41.5)	221 (36.8)
21,000-40,000	109 (54.5)	154 (38.5)	263 (43.8)
41,000-60,000	27 (13.5)	44 (11.0)	71 (11.8)
>61,000	9 (04.5)	36 (09.0)	45 (07.5)
Marital status			
Single	83 (41.5)	171 (42.8)	253 (42.3)
Married	105 (52.5)	196 (49.0)	301 (50.2)
Widow	6 (03.0)	20 (05.0)	26 (04.3)
Divorced	6 (03.0)	13 (03.2)	19 (03.2)
Family system			
Nuclear	89 (44.5)	161 (40.3)	250 (41.7)
Joint	111 (55.5)	239 (59.8)	350 (58.3)
Number of children			
1	6 (03.0)	23 (05.8)	29 (04.8)
2	17 (08.5)	30 (07.5)	47 (07.8)
3	36 (18.0)	38 (09.5)	74 (12.5)
4	19 (09.5)	39 (09.8)	58 (09.7)
5	25 (12.5)	24 (06.0)	49 (08.2)
6≤	4 (02.0)	37 (09.2)	41 (06.8)

^∗^The value in the parenthesis is the percentage of the adjusted frequency.

**Table 2:** Classification accuracy of training and testing part of artificial ANN (1, 4, 6) model.

Sample	Observed	Prediction		Percent correct
		OCD case	Control	
Training	OCD case	133	6	95.5%
	Control	5	285	98.3%
	Percent correct	32.2%	67.8%	97.4%

Testing	OCD case	58	1	98.3%
	Control	2	110	98.2%
	Percent correct	35.1%	64.9%	98.2%

Here, OCD stands for obsessive-compulsive disorder and ANN stands for Artificial Neural Network.

## Data Availability

The data used to support the findings of this study are available from the corresponding author upon request.
